# Advances in astrocytes in aneurysmal subarachnoid haemorrhage

**DOI:** 10.3389/fsurg.2025.1511242

**Published:** 2025-05-30

**Authors:** Jiahui Liu, Kun Sun, Qiushi Chen, Lianshu Ding

**Affiliations:** Department of Neurosurgery, The Affiliated Huaian NO.1 People's Hospital of Nanjing Medical University, Huaian, Jiangsu, China

**Keywords:** astrocytes, aneurysmal subarachnoid haemorrhage, subarachnoid haemorrhage complications, aSAH, review

## Abstract

Aneurysmal subarachnoid haemorrhage (aSAH) represents a subtype of stroke with a high incidence of morbidity and mortality. Astrocytes, the most abundant cell type in the central nervous system, play a pivotal role in brain injury and recovery following aSAH. They participate in synaptic remodelling, blood-brain barrier injury and the activation of the glial-mesenchymal-cervical lymphatic system. In this paper, we review the physiopathological functions and pathological changes of astrocytes after aSAH, exploring their mechanism of action in aSAH. We also summarise the evidence of therapeutic approaches to modulate astrocyte function after aSAH complications, and provide some new clues for future translational therapies to mitigate injury after aSAH.

## 1 Introduction

Aneurysmal subarachnoid haemorrhage (aSAH) is a complex cerebrovascular disease with a high incidence of morbidity and mortality worldwide. The global incidence of aSAH is approximately 700,000 cases per year, with an overall mortality rate of approximately 40% (1). Despite advances in aneurysm interventional embolisation and aneurysm clamping techniques, brain injury after aSAH is multimodal, with common complications of cerebrovascular spasm (CVS) and delayed cerebral ischaemia (DCI). Of these, DCI remains the most common cause of morbidity and mortality after initial treatment of ruptured aneurysms. The morbidity and mortality rates among surviving patients (2). For decades, CVS has been identified as a significant contributor to the adverse outcome of aSAH. However, the results of clinical trials investigating the palliation of CVS have been inadequate. In light of the aforementioned, there is an urgent need for further research into alternative mechanisms of brain injury following aSAH.

Astrocytes are widely distributed throughout the central nervous system (CNS) as neural precursor cells. They were initially described by Rudolph Virchow in 1858 as a form of connective tissue that holds neurons together, akin to “glue” in its adhesive properties. Indeed, in a healthy brain, astrocytes provide support and nutrition for neurons (uptake of glutamate to regulate neuronal metabolism), participate in the formation and signalling of neuronal synapses (the physiological space between signalling neurons where neurotransmission occurs), maintain the structural and functional stability of the blood-brain barrier(BBB) (the protective cell layer that protects the vasculature in the CNS), and improve cerebral microcirculation (maintaining the extracellular environment through ionic and water channels). Furthermore, astrocytes facilitate the maintenance of the extracellular environment, regulate cerebral blood flow, and stabilise cell-cell communication. They also provide nutrients and energy to brain tissues (3).

In recent years, there has been a significant increase in research attention devoted to astrocytes in a variety of brain injury and brain disease models. In the initial stages of aSAH, astrocytes undergo pathological activation, transforming into reactive glial cells and thereby initiating the subsequent inflammatory response. The involvement of astrocytes in the pathological process of aSAH remains incompletely understood (4).

## 2 Physiological and pathological functions of astrocytes

### 2.1 Astrocyte

Astrocytes have their origin in neural progenitor cells in the subventricular zone and migrate along radial glial cell protrusions to populate all areas of the CNS (5). Similarly to microglia, astrocytes exhibit both resting and reactive states (6). In the absence of pro-inflammatory stimuli, these cells are involved in several key processes related to CNS homeostasis. These include neuro-transmitter recirculation, ionic homeostasis, regulation of synaptogenesis and synaptic transmission, and maintenance of the BBB (7).

### 2.2 Reactive astrocyte

In response to pathological conditions, astrocytes frequently undergo a process known as “reactive astrocytosis,” which encompasses functional, morphological, and molecular remodeling (6, 8, 9). Reactive astrocytes are highly heterogeneous, and recent molecular techniques (single-cell-based RNA sequencing and influencing the function of astrocytes in CNS inflammation or degeneration) have identified astrocyte subpopulations: the developmentally induced astrocyte (DIA) subpopulation and the stimulus-induced astrocyte (SIA) subpopulation. These subpopulations may reflect at least two sources of heterogeneity in astrocytes (3). Reactive glial cells display distinctive morphological alterations, including an increase in cell body size and an elevation in the expression of the glial fibrillary acidic protein (GFAP) marker (8, 10). Moreover, reactive astrocytes undergo notable morphological alterations, including cell body hypertrophy and an increase in GFAP expression (11, 12). Additionally, the functions of reactive astrocytes differ from those of normal astrocytes, as they play crucial roles in axonal growth and synaptic remodelling, glial scar formation, regulation of BBB permeability, mobilisation of progenitor cells and immunomodulation.

## 3 Astrocyte molecular expression changes induced by aSAH

**Figure 1:**
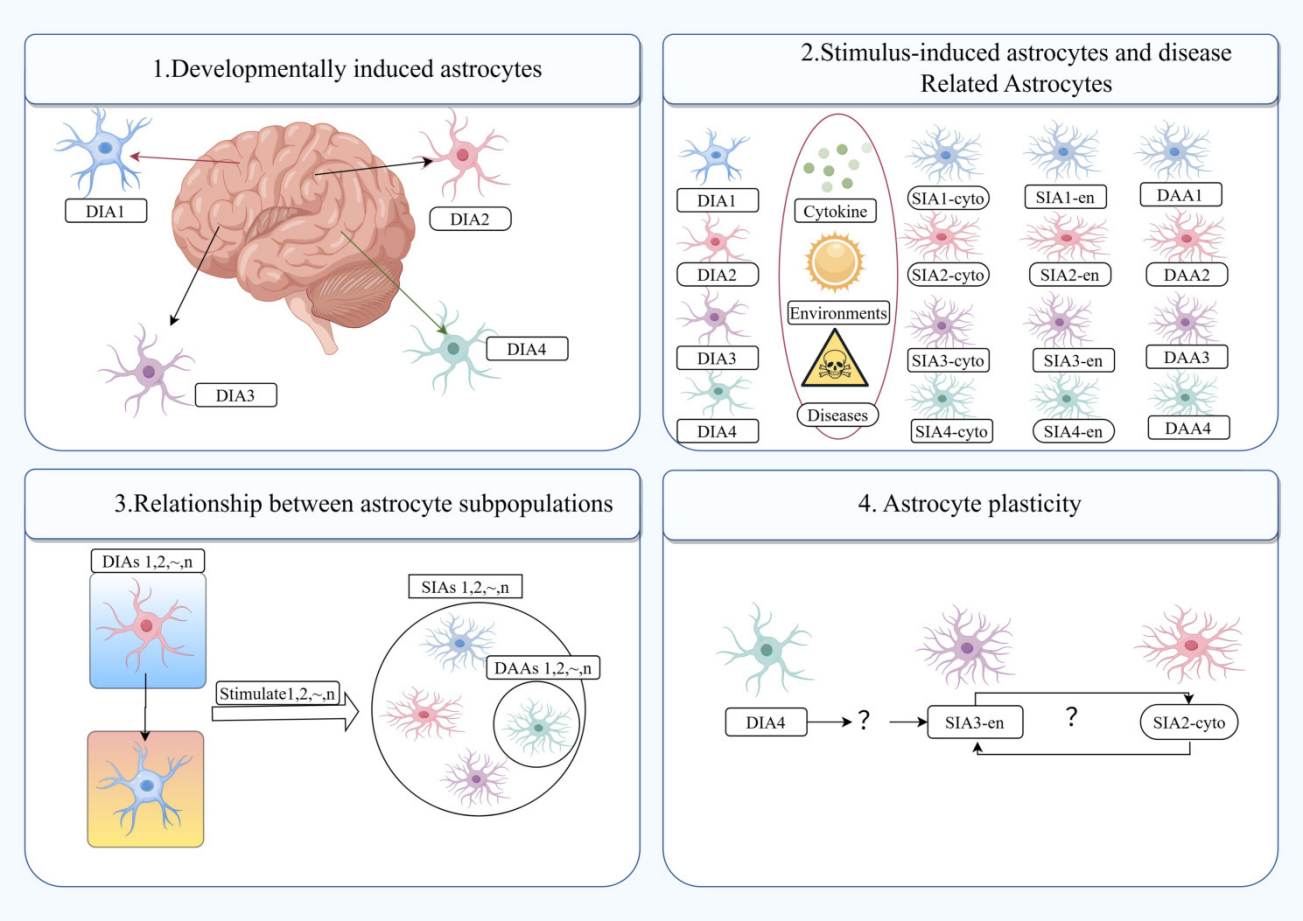
Newly defined subcomponent phenotypes of astrocytes. DIA, developmentally induced astrocyte; DIAs, developmentally induced astrocyte subpopulations; SIA, stimulus induced astrocyte; SIAs, stimulus induced astrocyte subpopulations; DAAs, disease-associated astrocytes; cytokines, environmental factors and diseases can induce SIAs from various DIAs, shown here as SIA1-4cyto, SIA1-4en and DAA1-4.

Jha et al. have put forth the notion that there exist two distinct states of reactive astrocytes: A1 and A2. The newly defined subcomponent phenotypes of astrocytes are shown in Figure 1. They posit that astrocytes in the A1 state exhibit neurotoxicity induced by inflammatory factors, whereas those in the A2 state demonstrate neuroprotective properties by upregulating neurotrophic factors in ischemic injury (11, 13). In the initial stages of aSAH, astrocytes undergo a transformation, becoming reactive astrocytes with an increased expression of GFAP. GFAP is a principal component of astrocyte intermediate filaments and is the most commonly utilised marker for reactive astrocytes. In addition to GFAP, clinical observations have identified calcium-binding protein B (S100B) as a novel biomarker of aSAH. In patients with aSAH, elevated levels of S100B in cerebrospinal fluid (CSF) have been linked to diminished 1-year clinical outcomes (14). Astrocyte polarization towards A1 and A2 phenotypes has been observed in a rat model of aSAH (12). Concurrently, S100 B has been demonstrated to stimulate the secretion of IL-6 and TNF-α in cultured astrocytes, exhibiting a concentration and time-dependent effect. This suggests a potential role for S100 B in the pathogenesis of SAH. Additionally, several aSAH-induced molecular changes in reactive astrocyte expression have been documented and are summarised in Table 1.

**Table 1:** Molecular changes in astrocytes aSAH.

Molecular changes in astrocytes	Define	Conclusion	Bibliography
GFAP	TypeIII intermediate filamentous protein	Markers of reactive astrocytes	(15)
ET-1	Vasoconstrictor mediator-1	The higher the expression level, the higher the risk of DCI	
AQP4	Aquaporin4	Elevated levels have been linked to the development of cerebral oedema, hydrocephalus and DCI	(16)
MMP-9	Matrix metalloproteinase-9	Destruction of the BBB	(17)
S100B	Calcium-binding protein	High level of prediction of adverse long-term outcomes	(18)
GLT-1	Glutamate transporter protein-1	Downward adjustment after aSAH	(19)
HDAC2	Histone deacetylase 2	HDAC2 inhibition improves DCI	(19)
TLR3, TLR4	Toll-like receptor 3,4	Activation of TLR leads to pro-inflammatory cytokine synthesis	
HO-1	Heme oxygenase-1	Reducing neuronal cell death and cognitive impairment	

## 4 Mechanisms of astrocyte involvement following aSAH

**Figure 2:**
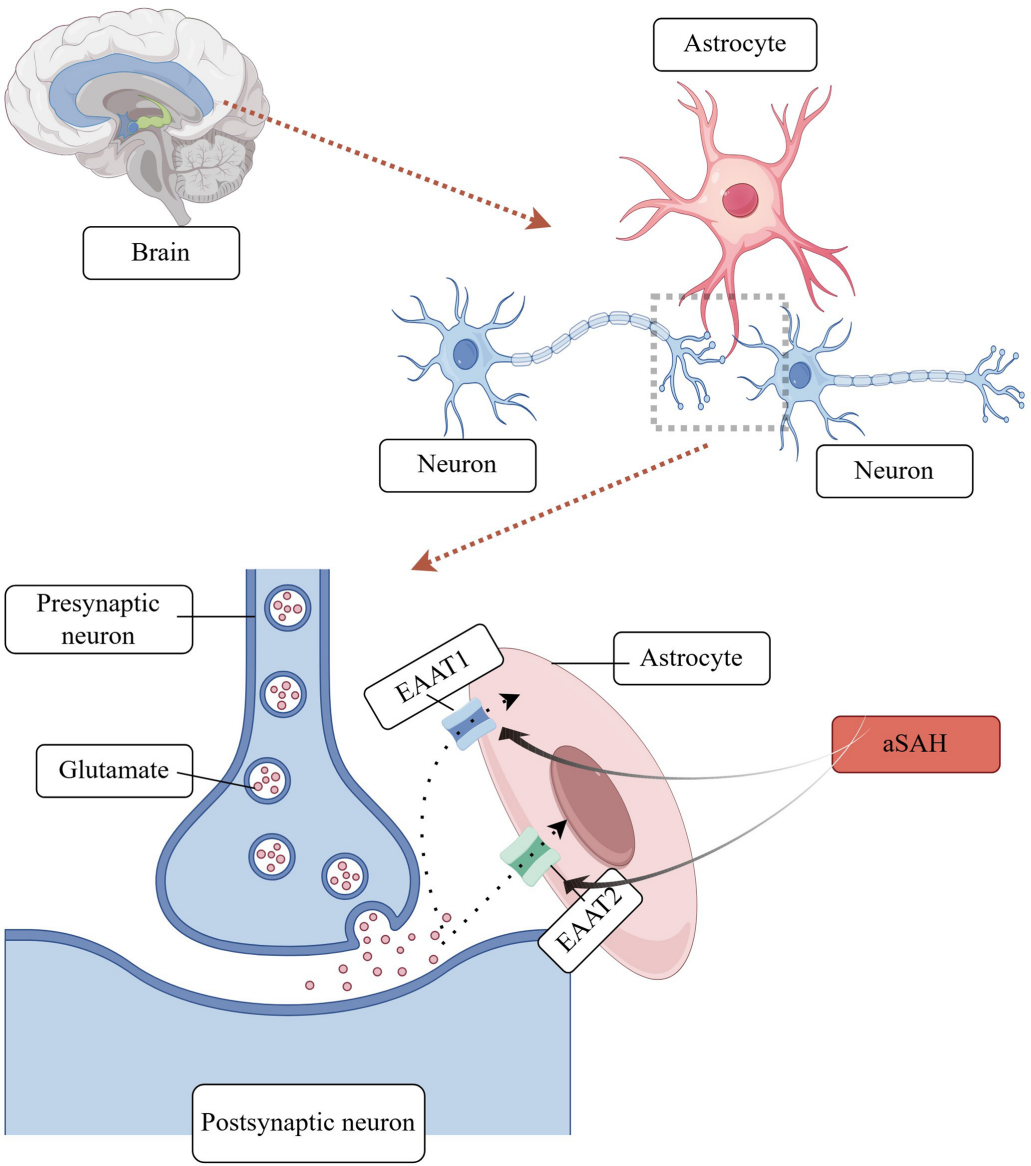
Astrocyte involvement in synaptic remodelling.

Synaptic remodelling: Glutamate is the primary excitatory neurotransmitter in the central nervous system, facilitating the transmission of information and contributing to processes such as learning, memory formation, and synaptic plasticity (20). Following aSAH, astrocytes are exposed to glutamate, which can exert an excitotoxic effect on these cells, thereby influencing their synaptic plasticity properties. aSAH elevates the extracellular concentration of glutamate. The elevated glutamate concentration is optimally maintained by the removal of glutamate from the synapse by astrocyte glutamate transporter proteins. In the human brain, five glutamate transporter proteins, or excitatory amino acid transporter proteins (EAATs), have been identified. Of these, two are expressed by astrocytes: EAAT 1 (GLAST) and EAAT 2 (GLT-1) (21). Recent studies have demonstrated that astrocytes, in addition to absorbing glutamate, also secrete minute quantities of this neurotransmitter to neighbouring neurons. This facilitates the synchronisation of neuronal firing and the regulation of excitatory or inhibitory synaptic transmission (22). A schematic diagram of astrocyte involvement in synaptic remodelling mechanisms is shown in Figure 2.

**Figure 3:**
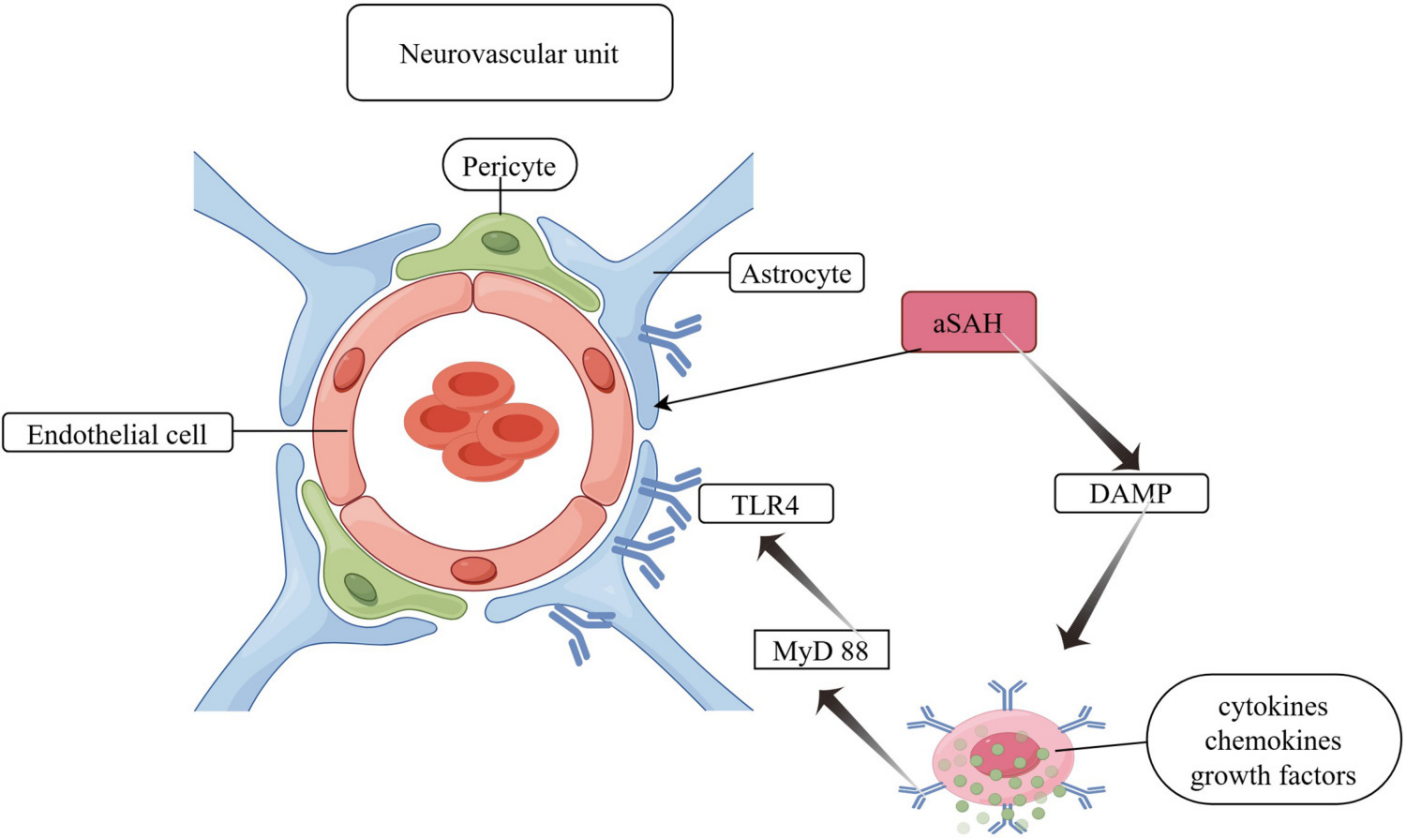
Astrocyte involvement in BBB disruption. DAMP, damage-associated molecular pattern; TLR 4, toll-like receptor 4; MyD 88, myeloid differentiation primary response protein 88.

BBB disruption: The BBB is constituted by a variety of interacting cells, including neurons, astrocytes, microglia, pericytes, endothelial cells and vascular smooth muscle cells. These include pericytes situated between the endothelium and the basement membrane, neurons, astrocyte end-feet and microglia, which collectively constitute the neurovascular unit (NVU) (23, 24). All NVU components contribute to the maintenance of a stable and functional BBB. The NVU components interact with each other and are able to establish different ionic microenvironments in the CNS, thus ensuring stable neuronal function. This is achieved by reducing cell proliferation, preventing toxin invasion and regulating inflammatory cell passage through the barrier to avoid the inflammatory process. Astrocyte deformations after aSAH include deformations of pedunculated synapses anchored to the basement membrane, which lead to ultrastructural disruption of the brain (25). Furthermore, it responds to damage-associated molecular patterns from the perivascular space, promoting the expression of pro-inflammatory cytokines, chemokines, and growth factors, as well as the recruitment and activation of peripheral immune cells (26). The increased expression of TLR 4 (Toll-like receptor 4) in astrocytes, which resulted in a worsening of neuroinflammation, was confirmed by the overexpression of myeloid differentiation primary response protein 88 (MyD 88). Activation of the TLR 4/MyD 88 pathway on days 1 and 5 after aSAH induction resulted in the ubiquitination of tumour necrosis factor receptor-associated factor 6 (TRAF 6). It is postulated that ubiquitination of TRAF 6 may increase the degradation of ULK 1 (a cytosolic autophagy enzyme), which may inhibit autophagy and exacerbate brain damage after aSAH (23). A schematic diagram of astrocyte involvement in BBB disruption mechanisms is shown in Figure 3.

**Figure 4:**
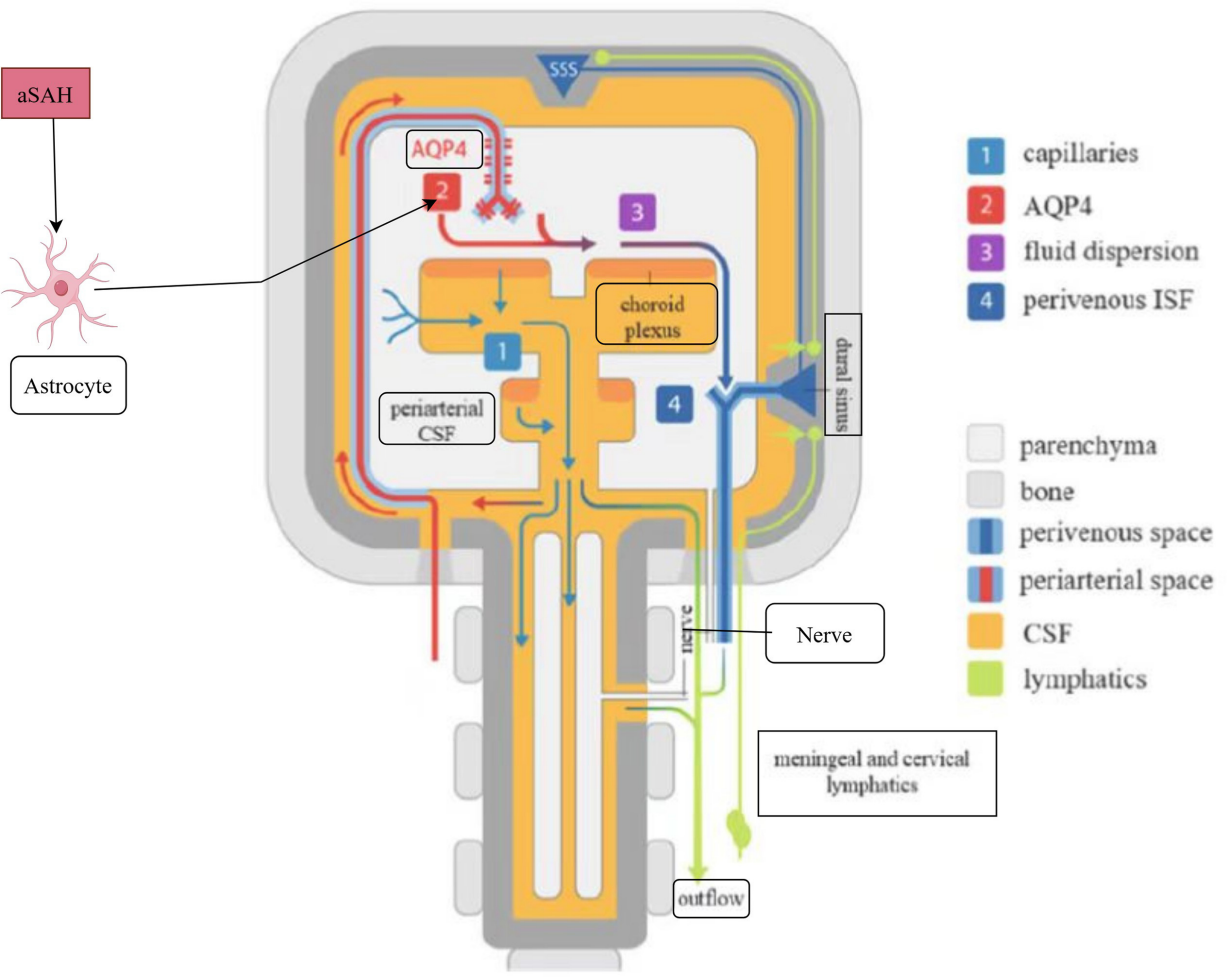
Astrocyte involvement in activation of the glial-membranous-cervical lymphatic system.

Activation of the glial-membranous-cervical lymphatic system: In October 1974, Helen F. Cserr's team posited the hypothesis that the perivascular space of the brain may serve as a conduit for the exchange of CSF and interstitial fluid (ISF). This proposition underscores the notion that the movement of CSF is propelled by a continuous, cyclical process driven by its production in the choroid plexus (27). In August 2012, Jeffrey J. Iliff's team provided further clarification on the pathways of mouse CSF. They determined that the majority of CSF enters the brain parenchyma along the paravascular space surrounding the periarterioles and is subsequently channelled out of the neck through the paravascular space. This space is dependent on AQP4 in the end-foot of astrocytes for its functionality. Additionally, the team observed that this space serves a similar role to the lymphatic system in terms of clearance. Consequently, this system has been named the “glial-lymphatic (Glymphatic) system” (27). In August 2022, the team led by Jean-Leon Thomas discovered that the intracranial meningeal lymphatic system is connected extracranially to the cervical lymphatic system via the cavernous sinus. Furthermore, they found that CSF can be drained from the superficial and deep cervical lymph nodes through this channel (28). A substantial corpus of literature posits that the aggregation of AQP4 channels within the astrocyte end-foot is the primary mechanism responsible for the generation of interstitial fluid that flows through the glial lymphatic pathway (29–31). Following aSAH, two opposing effects have been observed. On the one hand, intracranial residual blood can be drained through the glial-membrane-cervical lymphatic system, which may alleviate the neurological damage. On the other hand, the activated glial-membrane-cervical lymphatic system up-regulates the level of AQP4, which may lead to the development of cytotoxic cerebral oedema. It is therefore recommended that further study be conducted into the double-edged sword effect of AQP4 changes in pathophysiology after aSAH. A schematic diagram of astrocyte involvement in activation of the glial-membranous-cervical lymphatic system is shown in Figure 4.

## 5 Astrocytes in post aSAH complications and therapeutic value

Below is a summary of the role of astrocytes in complications following aSAH (see Table 2).

**Table 2:** Secondary complications of aSAH.

Common secondary complications	Relationship with astrocytes	Outlook
Hydrocephalus	Astrocyte swelling associated with vasogenic oedema	It is possible that early brain injuries may have a greater impact than interventions targeting vasospasm or DCI
Cerebral vasospasm	Astrocytes predominantly express ET-1 and ET-1 is essential in the initiation and maintenance of CVS	Nimodipine is still used, and animal studies have demonstrated that synthetic and semi-synthetic molecules as well as herbal substances can target astrocytes to relieve vasospasm (25)
Increased intracranial pressure, brain herniation	Associated with cerebral oedema and cerebral haemorrhage	Mannitol dehydration and surgery
Hydrocephalus	Increased total amount of AQP4 expressed by astrocytes and decreased polarity of AQP4; regulation of inflammatory factors and oxidative stress	The objective is to develop drugs that act on astrocytes to clear neuroinflammation and to target astrocyte AQP4 expression in order to address cerebrospinal fluid
Delayed cerebral ischaemia	Astrocytes combined with microglia may be able to both sense and induce extended cortical spreading depressin	DCI has a delay in the onset of aSAH, which may allow for a window of opportunity following aSAH during which neuroprotective interventions can be initiated
Delayed cognitive impairment	An increase in HDAC 2 expression in astrocytes results in the inhibition of histone deacetylation and GLT-1 expression, which in turn leads to a reduction in capillary coverage in the endfeet of AQP4-positive astrocytes	Ceftriaxone and targeted agents against AQP channels

Astrocytes and cerebral oedema: Cerebral oedema represents a significant element of the initial brain injury following aSAH. The prevailing view is that astrocyte swelling in aSAH is linked to the development of vasogenic oedema (caused by extravasation of plasma proteins and accumulation of fluid in cerebral oedema). AQP4 is the primary water channel protein expressed in astrocytes and plays a crucial role in maintaining water homeostasis within the BBB. aSAH is characterised by a significant surge in the activation of AQP4 and the cation channel protein sulphonylurea receptor 1, leading to the formation of an ionic/aqueous channel complex. This complex impairs the normal lymphatic circulation of cerebrospinal fluid (32). Consequently, the surgical obstruction of cerebral lymphatic drainage following aSAH serves to exacerbate the formation of cerebral edema (17).

Astrocytes and cerebral vasospasm: Complications of aSAH, such as CVS, are a significant concern in the post-aSAH period, typically emerging within 3–15 days. Following aSAH, astrocytes predominantly express ET-1, which plays a pivotal role in the initiation and maintenance of CVS. The increased expression of ET-1 results in contraction of vascular smooth muscle cells (VSMC), while stimulation of ET-1 receptors activates protein kinase C (PKC) and Ras homolog A (RhoA)/Rho kinase, leading to increased phosphorylation of myosin light chain phosphatase (MLCP). This ultimately results in the development of CVS. Consequently, ET-1 receptor antagonists are efficacious in the prevention and alleviation of SAH-induced CVS. Pro-inflammatory cytokines, growth factors and oxygen free radicals induce VSMC de-differentiation, thereby causing the so-called phenotypic transformation which leads to CVS. The mammalian target of rapamycin (mTOR) and proliferating cell nuclear antigen (PCNA) may play a role in the regulation of vascular smooth muscle cell (VSMC) growth, proliferation, survival, and protein synthesis. The increased expression of mTOR and PCNA in VSMC contracting at seven days after aSAH induction lends support to this suggestion (33).

Astrocytes and hydrocephalus: It is estimated that between 12% and 31% of patients who have experienced an aSAH will go on to develop hydrocephalus. Astrocytes have the capacity to regulate the aberrant expression of water channel proteins, which can in turn lead to the development of hydrocephalus. Sprague-Dawley rats have been observed to exhibit increased mRNA expression of water channel proteins 1, 4, and 9 in a model of kaolinite-induced hydrocephalus. Additionally, clinical evidence suggests that AQP4 overexpression and glial lymphatic damage are observed in surviving patients with idiopathic normal pressure hydrocephalus (iNPH), along with astrocyte proliferation (34, 35). As previously described, the total amount of AQP4 expressed by astrocytes increased and the polarity of AQP4 decreased after aSAH. Conversely, astrocytes possess the capacity to regulate inflammatory factors and oxidative stress, predominantly through the modulation of nuclear factor-κB (NF-κB) and IL-1β, which may contribute to the development of hydrocephalus. At present, the primary treatment for hydrocephalus is surgical intervention. However, studies investigating non-surgical alternatives have yielded unsatisfactory results (36). In light of the aforementioned analysis of the role of astrocytes in hydrocephalus following aSAH, future research may focus on developing drugs that target astrocytes to reduce neuroinflammation, regulate astrocyte AQP4 expression to address cerebrospinal fluid issues, and utilize specific antioxidants to scavenge free radicals.

Astrocytes and DCI: DCI is a common occurrence following aSAH and is regarded as a significant contributor to long-term dysfunction. CVS has long been considered a significant contributing factor to DCI, with DCI often resulting in cerebral infarction. Below is a list of complications related to aSAH that are easily confused; specific differentiation can be found in Table 3.

**Table 3:** Recommended terms and definitions for late complications of aSAH.

Recommended terminology	Other terms	Definition
Angiographic vasospasm	Vasospasm, arterial stenosis, radiological vasospasm	Cerebral artery stenosis detected by angiography such as DSA, CTA or MRA
Delayed cerebral ischaemia	Delayed ischaemic neurological deficit, delayed ischaemic deficit, delayed neurological deficit, secondary cerebral ischaemia, vasospasm, clinical vasospasm, symptomatic vasospasm, symptomatic ischaemia	Clinical symptoms of ischaemia or imaging (CT/MRI) evidence of ischaemia, quantitatively defined as the occurrence of focal neurological deficit (e.g., hemiparesis, aphasia, dysarthria, hemianopsia, or neglect), or a decrease in GCS of at least 2 points. Symptoms persisted for ≥1 h by clinical assessment, cranial CT or MRI, and laboratory tests, did not occur immediately after aneurysm occlusion, and could not be attributed to other causes
Cerebral infarction	Low density stove	The presence of a cerebral infarction on cranial CT or MRI within 6 weeks of the onset of SAH, or on the most recent CT or MRI prior to death within 6 weeks, or confirmed at autopsy, is not present on CT or MRI 24–48 h after early aneurysm occlusion and is not attributable to other causes such as surgical clipping or endovascular therapy. CT hypodense shadows due to extraventricular drains or intracerebral parenchymal haematomas should not be considered as cerebral infarction due to DCI

However, recent studies have concluded that microarterial dysfunction, microthrombosis, cortical spreading depolarization and neuroinflammation are closely related to DCI and the development of cerebral infarction. Furthermore, these mechanisms are not independent of each other, but rather are interconnected and work together to lead to DCI. Microvascular constriction within the CNS parenchyma provides support for the prethrombotic environment, whereas cortical spreading depolarization has the potential to disrupt neurovascular coupling and induce vasoconstriction. It is possible that activated astrocytes in association with microglia may be able to both sense and induce cortical spreading depolarization, thereby further exacerbating injury by promoting imbalances in ionic homeostasis and synaptic pruning (37). In addition to the aforementioned mechanisms, impaired cerebrospinal fluid flow and waste removal from the meningeal lymphatic system can also result in DCI and cerebral infarction. In terms of therapeutic options, oral nimodipine remains the primary drug employed to prevent DCI. However, there is also considerable interest in goal-directed haemodynamic therapy and milrinone (38).

Astrocytes and delayed cognitive impairment: Delayed cognitive dysfunction following aSAH represents a relatively common adverse outcome in the later stages of aSAH, with a significant impact on patients' quality of life. Astrocytes in the hippocampus display enlarged cell bodies and retracted protrusions, leading to a reduction in capillary coverage of the astrocyte endfeet. Morphological alterations in hippocampal astrocytes impair astrocyte-capillary interactions, thereby contributing to the pathogenesis of long-term cognitive dysfunction following aSAH. Conversely, the reduction in Akt phosphorylation resulting from aSAH has been observed to result in a decline in the expression of EAAT-2 in astrocytes. The administration of ceftriaxone, an antibiotic that has been demonstrated to up-regulate the expression of EAAT-2, has been shown to enhance glutamate uptake via EAAT-2, a process that could potentially be harnessed for the treatment of cognitive impairment following aSAH (39). Following aSAH, increased HDAC 2 expression in astrocytes results in histone deacetylation and the inhibition of GLT-1 expression. This leads to a prolonged accumulation of glutamate in the synaptic gap, which in turn causes dephosphorylation of the ionotropic glutamate receptors, GluA 1 and GluN 2B, on the postsynaptic membrane. Such alterations may exert a detrimental influence on hippocampal synaptogenesis, ultimately resulting in cognitive impairment.

## 6 Discussion

Despite decades of exploration, the prognosis of aSAH remains far from satisfactory. This review presents a summary of the subpopulations of astrocyte reactivity, their activation during aSAH, and their involvement in prominent remodelling, disruption of the BBB, and the lymphatic-membrane lymphatic system. Additionally, it addresses their involvement in post-aSAH complications and provides an overview of potential drugs targeting astrocytes.

While the number of human studies on astrocytes in aSAH is limited, discussing these studies is essential to connect preclinical findings with clinical relevance. For example, research indicates that elevated S100B levels in CSF correlate with poor clinical outcomes in aSAH patients. As a calcium-binding protein and astrocyte activation biomarker, S100B may serve as a potential therapeutic target, highlighting the significant role of astrocytes in aSAH pathophysiology. Milrinone, a phosphodiesterase inhibitor, has been widely used in heart failure treatment. Recent studies suggest it may also alleviate cerebral vasospasm. This discovery could offer a novel therapeutic approach for complications following aSAH (23).

Preclinical studies have demonstrated that astrocytes may exert beneficial effects in reducing neuronal damage and cognitive deficits following aSAH. There is an urgent need to develop treatments that improve the prognosis of patients with aSAH. What methodology can be employed to identify the optimal astrocyte subpopulations for the purpose of elucidating the pathogenesis of aSAH and identifying potential therapeutic targets? To address this, we propose the use of advanced molecular profiling techniques, such as single-cell RNA sequencing (scRNA-seq), to identify specific astrocyte subpopulations involved in aSAH pathology. Additionally, the application of *in vivo* imaging techniques could help track the dynamics of these subpopulations in real-time, providing valuable insights into their roles in disease progression. The development of emerging technologies has enabled the identification of local, microbial, and environmental factors controlling different subpopulations of astrocytes. For example, scRNA-seq has been used to identify distinct astrocyte subpopulations with unique molecular signatures in various neurological diseases. In aSAH, these technologies could help elucidate the heterogeneity of astrocyte responses and identify potential therapeutic targets. Furthermore, the use of optogenetic and chemogenetic tools could allow for precise manipulation of specific astrocyte subpopulations, helping to clarify their functional roles in disease mechanisms (40).

**Figure 5:**
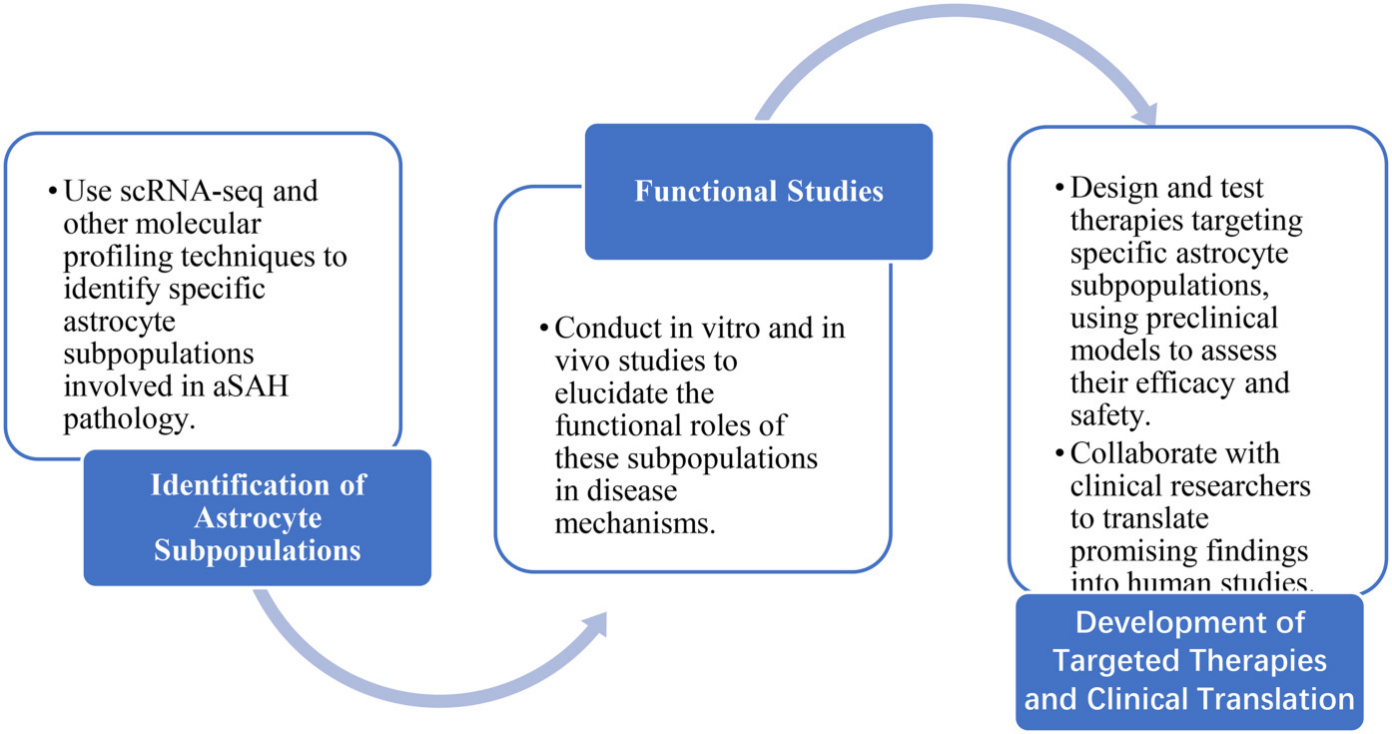
Research roadmap.

However, it is important to acknowledge its limitations. The majority of the evidence discussed is derived from animal models, which may not fully recapitulate the complexity of human aSAH. Furthermore, the heterogeneity of astrocyte responses and the lack of specific biomarkers for different astrocyte subpopulations pose significant challenges for targeted therapies. Future research should focus on validating findings in human studies and developing more specific targeting strategies. To advance our understanding of astrocytes in aSAH and develop effective therapies, we propose the research roadmap shown in Figure 5.

## 7 Conclusion

In conclusion, this review has examined the diverse roles of astrocytes in the pathophysiology of aSAH. We have elaborated on the physiological and pathological functions of astrocytes, emphasizing their contributions to synaptic remodelling, BBB compromise and glial-lymphatic system activation following aSAH. The evidence highlights the critical involvement of astrocytes in the development and modulation of secondary complications after aSAH, including cerebral edema, vasospasm, hydrocephalus, delayed cerebral ischemia, and cognitive deficits. Moreover, we have explored the therapeutic potential of targeting specific astrocyte subpopulations and molecular pathways, which could provide innovative strategies for reducing injury and enhancing outcomes in aSAH patients. Future research should concentrate on pinpointing the optimal astrocyte subpopulations for targeted therapies and conducting clinical trials to evaluate the safety and efficacy of these approaches in human subjects.
